# Kuhnian revolutions in neuroscience: the role of tool development

**DOI:** 10.1007/s10539-018-9628-0

**Published:** 2018-05-04

**Authors:** David Parker

**Affiliations:** Department of Physiology, Development and Neuroscience, University of Cambridge, Cambridge, CB2 3DY UK

**Keywords:** Neuroscience, Kuhn, Paradigm, Paradigm shift, Revolution, Optogenetics

## Abstract

The terms “paradigm” and “paradigm shift” originated in “The Structure of Scientific Revolutions” by Thomas Kuhn. A paradigm can be defined as the generally accepted concepts and practices of a field, and a paradigm shift its replacement in a scientific revolution. A paradigm shift results from a crisis caused by anomalies in a paradigm that reduce its usefulness to a field. Claims of paradigm shifts and revolutions are made frequently in the neurosciences. In this article I will consider neuroscience paradigms, and the claim that new tools and techniques rather than crises have driven paradigm shifts. I will argue that tool development has played a minor role in neuroscience revolutions.

In “The Structure of Scientific Revolutions” (SSR; Kuhn 1962), Thomas Kuhn outlined his view of how science was done, rather than how it should be done. The SSR introduced several terms, principally “paradigm” and “paradigm shift” that are used routinely by scientists. However, it is unlikely that many scientists would consider themselves “Kuhnian”. Kuhn suggested that most accounts of science (e.g. textbooks, reviews) are hindsight revisions (see also Medawar 1964), which give the impression of a logical and cumulative progression towards truth (i.e. the view that most scientists would promote). Kuhn also claimed that scientists learn by immersive training, getting what Michael Polanyi called “tacit knowledge” (Polanyi 1966). This views science as something practiced, where disciplined minds learn the rules and habits of a field. Kuhn said that science offered a narrow and rigid education “probably more so than any other except perhaps in orthodox theology”.

Kuhn claimed that the science practiced by a field was governed by a paradigm. This resembled the earlier “thought collective” of Ludwik Fleck (see Fleck 1979), the mutual ideas that determine the “thought style” of researchers, and Abraham Maslow’s “means-centred” science, the tools and techniques learnt during a scientific education (Maslow 1946). A paradigm develops from a pre-paradigm state that lacks a dominant idea, and once established provides the knowledge learnt during scientific training. A paradigm is incomplete, its gaps providing the “puzzles” that occupy “normal science”, the questions asked and the work done under a paradigm. The paradigm guarantees solutions to these puzzles, but it takes effort and ingenuity to find them. Kuhn claimed that competence in finding solutions that fit with a paradigm determines an individual’s scientific credibility.

A scientist also has to reconcile anomalies, aspects that don’t seem to fit with the paradigm. Under a paradigm the expectation is that anomalies will eventually be reconciled with the paradigm. However, this can be difficult, and requires commitment to the paradigm. Kuhn used the failure of the Newtonian paradigm to explain the anomalous orbit of Uranus to illustrate how this commitment can address anomalies: rather than claim an error in the Newtonian paradigm, Le Verrier and Adams independently predicted that an unknown planet must influence Uranus’s orbit, ultimately leading to the discovery of Neptune. A recent example is the CERN announcement that neutrinos exceeded the speed of light (OPERA collaboration 2012). The special theory of relativity was not rejected following this announcement, commitment to the paradigm ensuring that the anomalous result was greeted with caution and found to be in error. Kuhn’s outline of paradigms led to claims that he was a relativist, but he did not see normal science as negative or irrational, but as necessary to allow scientists to work and communicate effectively. Kuhn highlighted how Karl Popper’s falsification method, which is often presented as “the” scientific method, would in its naïve form (Lakatos 1970) leave science in chaos, every error or anomaly leading to hypotheses being rejected.

Despite being a key aspect of the SSR, Kuhn’s definition of a paradigm was initially unclear. Masterman (1970) counted 21 uses of the word. She organised these into three groups: the metaphysical (beliefs, standards, or speculations); the sociological (universally recognised scientific achievements); and the construct (the tools, techniques, methods, or approaches that direct research). In response, in the 2nd edition of the SSR (1970), Kuhn distinguished between a broad concept of a paradigm, the disciplinary matrix (aspects that bind a community together including formal theories and definitions), and the narrower concept of the exemplar (e.g. individual solutions to problems, or specific methods and techniques).

Kuhn claimed that normal science is periodically interrupted by a scientific revolution caused by a crisis in the paradigm. A crisis develops when a paradigms accuracy or usefulness diminishes, or anomalies increase in number or significance that despite effort cannot be addressed by the paradigm. During a crisis there may be attempts to resist change, especially by those strongly associated with the paradigm: an anomaly can be ignored by claiming it was an error or the scientist who identified it was biased or incompetent; by direct or tacit coercion to prevent anomalies being reported (e.g. using peer review to block publication); and even claims that the paradigm is becoming more successful. This can lead to the absurdities and sense of disorder that characterises a crisis. The crisis is resolved by a “paradigm shift”, as a new paradigm replaces the previous paradigm in a scientific revolution.

In the first edition of SSR Kuhn claimed that old and new paradigms were incommensurate (mirroring Fleck’s claim that ideas could only be understood by those within a thought collective; see Fleck 1979). Thus, followers of the caloric account of heat wouldn’t explain their results in the same way as followers of mean kinetic energy, even if they used the same experiment, equipment, and words (e.g. ‘temperature’). Kuhn said that understanding was not gradual but reflected a “gestalt shift” that required reference to the whole. He later claimed that partial translation and communication was possible (Kuhn 2000) but that it was difficult for the defining concepts of a paradigm [the astrophysicist Subrahmanyan Chandrasekhar spent 5 years translating Newton’s Principia into a form modern physicists could understand (Chandrasekhar 1995), but in places the translation still fails (Smith 1996)]. Difficulty in communication between paradigms would thus be a hallmark of a scientific revolution.

The terms paradigm shift and revolution have become scientific clichés, despite Kuhn’s examples suggesting that revolutions are rare. He said that after publishing the SSR he was often asked if particular discoveries were revolutions or normal science (Ohm’s law was a revolution because it used terms that were defined differently to before, but the Joule–Lenz law of heat in a wire was normal science because it used existing concepts). He said that determining whether something was a revolution was difficult. It needed a detailed study of the area either side of the revolution, and it also depended on who the revolution was for: the Copernican heliocentric universe was a revolution for everyone, but Lavoisier’s discovery of oxygen was principally a revolution for chemists.

## Neuroscience revolutions and tool development

Kuhn’s analysis was based on the physical sciences. An obvious question is whether this structure applies to the biological sciences. This is claimed for Darwin and Wallace’s theory of evolution and the development of molecular biology following Watson and Crick’s determination of the structure of DNA. However, neither matches a Kuhnian revolution; the former because it wasn’t generally accepted until the modern synthesis with Mendelian genetics in the 1930s, and the latter because it did not overthrow a previous paradigm but developed from a pre-paradigm state (see Wilkins 2005; Strohman 1997). A better example of a biological paradigm shift is the germ theory of disease in the nineteenth century, which incommensurably replaced the previous miasma theory (Gaynes 2011).

The terms paradigm shift and revolution are frequently used in the neurosciences, often with respect to new techniques or tools. For example, Bickle (2016) claims that paradigm shifts and revolutions in neuroscience do not match Kuhn’s outline as they have not reflected crises and anomalies, but the development of new experimental tools (“In short: understanding tool development is the key to understanding real revolutions in actual neuroscience.”; Bickle 2016). The view that tools drive scientific revolutions was suggested by Freeman Dyson in “The Sun, The Genome and the Internet” (1999), following Peter Galison’s “Image and Logic” (1997) that analysed of the role of tools in twentieth century physics. However, Kuhn claimed that new methods and instruments increase precision and understanding within a paradigm, which would make tool development an aspect of normal science (Kuhn 1962). The difference could depend on definition, but in contrast to the term paradigm Kuhn’s definition of a revolution seemed clear; “a noncumulative developmental episode in which an older paradigm is replaced in whole or in part by an incompatible new one” (SSR, p. 92), that “is not only incompatible but often actually incommensurable with that which has gone before” (SSR, p. 103). A new technique may thus be revolutionary, but would only cause a paradigm shift if it directly led to a conceptual change.

The development of new tools is clearly vital to research. Galileo’s inclined plane was needed to investigate how a body moves under its own weight and the Atwood machine to test Newton’s second law of motion (Kuhn 1961). However, both tested existing theories and were thus normal science. The computer is an obvious modern example, but does it just allow existing paradigm questions to be addressed differently? Dyson (1999) says that, “If the tools are good, nature will give a clear answer to a clear question”. But a clear question and answer and an elegant or sophisticated tool or technique will not overcome conceptual errors. For example, Laplace used data from Delaroche and Berard’s novel technique that allowed measurement of pressure effects on the temperature of a gas to address the anomaly in the measured and predicted speed of sound. However, he applied the erroneous calorific theory of heat to erroneous experimental measurements, the two mistakes cancelling each other out to give close agreement between the predicted and measured speed of sound (Mendoza 1990).

Two techniques developed over the last 30 years are claimed to be neuroscience revolutions: molecular genetics (e.g. gene knock-outs) and optogenetics (Bickle 2016). In keeping with the revolutionary theme, it has been claimed that these will overthrow previous techniques (e.g. “the photon will progressively replace the electron”; Scanziani and Hausser 2009). But to have caused a revolution, at least as defined by Kuhn, these techniques must have caused a conceptual change rather than addressing questions within existing paradigms.

Optogenetics is consistently referred to as a revolutionary technique (a reasonable claim) that has or is causing a scientific revolution. For example, Bickle (2016) says “it is difficult to deny that optogenetics’ impact on neuroscience has already been revolutionary”; Häusser (2014) says that optogenetics has caused “a revolution in neuroscience”; and Knafo and Wyart (2015) refer directly to Kuhn in saying that optogenetics “represents a true paradigm shift”. Optogenetics has revolutionised experiments, but has it caused any conceptual change? For example, Oka and Zuker (2015) used optogenetics to study the subfornical organ (SFO), stating the results “reveal an innate brain circuit (that) probably functions as a centre for thirst control”. Press releases reported that the drinking circuit in the brain had been discovered, but the SFO was implicated in drinking behaviour from at least 1970, and has been a textbook staple since at least the early 1990s (e.g. Principles of Neural Science 3rd edition). We know its inputs and its output to the hypothalamus, and that SFO stimulation can trigger drinking. Oka et al. repeat the stimulation experiments using optogenetics. The advance is that they identified populations of excitatory and inhibitory neurons with opposite effects on drinking. This is not trivial, but is a step advance of an existing concept, not a paradigm shift. Secondly, a highly cited paper by Kravitz et al. (2010) used optogenetics to activate the direct and indirect pathways in the basal ganglia. This elegantly examined how these pathways affect behaviour, but it tested a concept known since the early 1990s (Alexander and Crutcher 1990) rather than addressed outstanding questions about basal ganglia circuitry (e.g. Cazorla et al. 2015; Graybiel 2005). Finally, Ramirez et al. (2013) examined the link between hippocampal long-term potentiation (LTP) and memory by optogentically creating a false memory in the mouse hippocampus. This impressive experiment satisfied a key criteria claimed to be needed to link LTP to memory that had previously been impossible (Jeffery 1997). This is again an example of puzzle solving within normal science. As with the other studies, this removes none of its significance, but it is not a scientific revolution.

This view is echoed by users of optogenetics. Replies to the question, “has there been a major breakthrough in our fundamental understanding” (see Adamantidis et al. 2015) ranged from a begging the question “not yet” (Hauser); to showing “what we already knew” (Turrigiano), “confirmatory” (Josselyn), and “not caused a true paradigm shift” (Malenka). Boyden, who helped develop optogenetic approaches writes, “no major paradigm shift in neuroscience has resulted from the use of optogenetic tools …. What optogenetics has done so far is make the study of circuits more tractable” (Boyden 2015). This again defines normal, not revolutionary science. This is not a critique of optogenetics: confirmatory experiments are necessary with any new tool to show that it works. Optogenetics, like any technique, may trigger a scientific revolution, or it could become an example of “Maslow’s hammer”, used because it is available rather than because it addresses fundamental questions [e.g. the optogenetic activation of spinal cord networks (Hagglund et al. 2013)].

Tools are one aspect of a paradigm (Mastermans’s construct or Kuhn’s exemplar). Tool development can address limitations of existing techniques, but this is again the puzzle-solving of normal, not revolutionary science. Molecular genetics and optogenetics are not scientific revolutions, as this requires a change in concepts and a degree of incommensurability between pre and post-technique paradigms. This hasn’t happened: optogenetic and molecular genetic studies use existing terms and concepts. This can be shown by comparing the LTP field at the start (Bliss and Collingridge 1993) and 20 years after molecular genetic approaches were introduced (Bliss and Collingridge 2013). Many of the same issues remain, addressed in the same language, and referring to the same concepts. Someone who left the LTP field pre-1990 would have no difficulty understanding the post-molecular field (as demonstrated in Lømo 2017).

## Neuroscience paradigms

To examine the role of tools in neuroscience revolutions requires consideration of neuroscience paradigms. It is hard to claim a single governing paradigm given the diversity of neuroscience areas (developmental, physiological, and psychological) and levels of analysis (molecular, cellular, cognitive, and behavioural), which ask different questions and use different techniques, approaches, and concepts. Specific paradigms include animal electricity, the neuron doctrine, chemical synaptic transmission, and cerebral localisation. These provide routine textbook accounts, have a set of concrete beliefs and principles (Masterman’s sociological or metaphysical definition of a paradigm and Kuhn’s disciplinary matrix), and specific tools, techniques, and methods (Masterman’s concrete paradigm and Kuhn’s exemplar). These paradigms will be used to address the role of tool development in neuroscience revolutions.

## Animal electricity

The paradigm that arguably cuts across all neuroscience areas is animal electricity. Electrical signalling underlies the development, plasticity, and pathology of all sensory, motor, and cognitive functions, and analyses of the nervous system rely on electrical activity (directly for electrophysiology and EEG, indirectly for calcium imaging and fMRI). The animal electricity paradigm replaced the previous animal spirits paradigm based on Aristotle’s views on the mind in De Anima (the psyche was the source of movement and sensation), and the views of Galen, who believed that animal spirits contained in the brain ventricles controlled behaviour (Rocca 1997). Just as Kuhn had problems following Aristotle’s physics, it is difficult to follow these views. While the Ptolemaic earth-centred universe was for a while accurate enough for practical uses like navigation and a calendar, Galen’s anatomy and physiology would have had little practical use. He claimed digested food entered the liver to form blood containing impure pneuma; this went to the right chamber of the heart where impurities were exhaled through the lungs; purified blood then moved through pores to the left side of the heart where it was imbued with pneuma breathed in through the lungs; blood then travelled to a net of arteries, the rete mirabile, at the base of the brain where it collected the highest form of pneuma, the animal or psychic spirits that then entered the ventricles to direct actions. Human dissections in the middle ages revealed discrepancies in Galen’s anatomy. Paracelsus, echoing Ibn al-Nafis, concluded that Galenic teachings were worthless (Ochs 2004; West 2008). However, Galenic doctrine persisted: Fernel’s “Natural Part of Medicine”, where the term physiology originated, followed Galenic teachings (Ochs 2004); Vesalius in De Corporis Humani Fabrica corrected numerous aspects of Galen’s anatomy but the role of animal spirits flowing from the ventricles remained (Vesalius viewed the cortex as a route for blood vessels to nourish deeper parts of the brain). Finally, Thomas Willis believed that the circle of arteries he identified at the base of the brain delivered animal spirits to the ventricles that then flowed along hollow nerve fibres to cause movement by inflating muscles (see Finger 1994).

The animal spirit paradigm, which now seems bizarre, persisted until the end of the eighteenth century, and determined the concepts and questions asked about the brain and action (e.g. where and how animal spirits were generated, stored, and released). In the seventeenth century, anomalies arose that argued against animal spirits: for example, contraction of isolated nerve muscle preparations (i.e. disconnected from the ventricles); no increase of muscle volume during contraction; and no swelling of the nerve above a ligation that should have blocked the flow of animal spirits. These anomalies were dismissed or explanations offered to defend the animal spirit paradigm. For Kuhn a revolution only occurs when an alternative paradigm is available. This started to develop in the eighteenth century. La Mettrie provided a materialistic account of the brain and mind in opposition to the dualism of Descartes in his book “Man a Machine” (1748; see Ochs 2004), while the developing interest in electricity in the latter half of the eighteenth century offered “animal electricity” as an alternative paradigm. Animal spirits proponents dismissed this view, highlighting the failure of nerve conduction with ligature (it was erroneously assumed that this would not stop electrical signals); the slow speed of nervous compared to electrical conduction; and the issue of how a potential difference and directed conduction could occur when the whole body is a moist conductor (see Piccolino and Bresadola 2013). The first accepted evidence for animal electricity was the electrical discharge from the fish Torpedo. This showed that an iso-conductive body could in principle use electricity, but this was considered an exceptional case by adherents of animal spirits (see Piccolino and Bresadola 2013). The paradigm shift needed unequivocal evidence of animal electricity. This was provided by Luigi Galvani’s frog experiments (see Piccolino 1997; Verkhratsky et al. 2006). When a metal wire was placed across the spinal cord, contractions occurred when the nerve to the femoral muscle was touched with a scalpel, completing a circuit that Galvani believed allowed electricity to flow out from the muscle. Alessandro Volta initially admired Galvani’s experiments, but expressed doubt when he showed that contraction could occur when two metals were placed on the nerve, negating Galvani’s (erroneous) claim that electricity flowed from the muscle. Volta defended the animal spirits paradigm by claiming that Galvani had incorrectly interpreted a simple irritant effect of electricity generated by two dissimilar metals. Even when Galvani showed that contractions occurred when the circuit was completed with the same metal, Volta insisted there must have been some unknown impurity that generated electricity. Galvani subsequently showed that contraction did not increase beyond a plateau level, and fatigued with repetitive stimulation, aspects inconsistent with Volta’s explanation; that folding the cut end of the nerve onto the muscle caused contraction, thus removing all external metals; and finally, that both legs contracted when the surface of a cut femoral nerve was placed in contact with the opposite nerve. Even the latter experiment (which Emil du bois Reymond called “the most capital experiment of electrophysiology”; Piccolino 1998) did not settle the argument. After Galvani’s death in 1798 (he had been stripped of his academic position for not accepting the new political authority), Volta’s status and power of authority and his ad hoc explanations of Galvani’s effects allowed the animal spirits paradigm to persist. However, Galvani’s experiments attracted followers, including Giovanni Aldini (Galvani’s nephew), who made a coherent theory of electrical excitation of biological tissues. Galvani’s work thus offered the alternative that allowed the anomalies of the animal spirit paradigm to be considered (see Piccolino 1997; Verkhratsky et al. 2006 for further details).

By the first half of the nineteenth century, Galvani’s animal electricity had won over most doubters. As a result, the entire concept of the nervous system changed incommensurately: muscles were not “inflated” but contracted; brain ventricles did not store animal spirits; and nerves were not hollow tubes carrying spirits, air, or aether. Determining how nerves generated electrical signals became the puzzles of normal science done under the animal electricity paradigm (Piccolino 1998). New instruments and techniques were needed. Oersted’s galvanometer was used by Leopold Nobili to make the first recording of a neural signal from the frog leg nerve. Nobili assumed this “intrinsic current” was due to cooling caused by evaporation from the nerve (Piccolino 1998), illustrating that technique alone does not guarantee correct identification of a mechanism. Carlo Matteucci interpreted the current between an intact and cut end of a muscle as coming from the muscle itself, and showed using piles of frog legs that its magnitude was proportional to the number of legs. He called this potential the “negative variation”. Du Bois Reymond improved measurement instrumentation with his rheotome, which allowed direct measurement of the negative variation, from which he developed the concept of the negative resting potential and its depolarisation during an action potential (Piccolino 1998). A major puzzle was the apparent infinite velocity of these signals, which Johannes Muller said were too quick to be measured. Hermann von Helmholtz made this measurement, again using new equipment. He said that it was the assumption that the velocity was infinite, not lack of technique, that had held the field back (Piccolino 1998), highlighting the need for conceptual rather than technical change. Julius Bernstein, using another technological innovation, the differential rheotome, proposed that the nerve was selectively permeable to potassium and that this generated the negative resting potential, with action potentials resulting from the breakdown of this selectivity that brought the membrane potential to zero (for discussion see Piccolino 1998). Charles Overton developed this idea, demonstrating that Na^+^ ions were required for the “negative variation”, and that excitation resulted from sodium exchange for potassium, predating Hodgkin and Huxley by almost half a century (Huxley wrote, “In retrospect, the sodium idea seems very obvious, and Hodgkin and I felt that we had been stupid not to think of it at once in 1939. I am sure that we would have done so if either of us had known the paper by Overton”; Huxley 2002a).

In 1939, Cole and Curtis confirmed some of Bernstein’s ideas, especially the increased conductance during an action potential (Cole and Curtis 1939), while Hodgkin and Huxley negated Bernstein’s claim that the membrane potential went to zero during an action potential by showing that it overshot zero. After the war, Huxley heard from August Krogh that he had used radioactive tracers to show Na^+^ entering and leaving the cell (see Huxley 2002b for historical details). This work ultimately ended in Hodgkin and Huxley’s demonstration that the action potential reflected increases in sodium and potassium permeability (Hodgkin and Huxley 1952). The development of the voltage-clamp technique was crucial to this work, together with the use of an appropriate model system, the squid giant axon.

Was Hodgkin and Huxley’s work revolutionary? Armstrong and Hille (1998) write, “The assumption of separable permeability components and the realization that membrane potential is the controlling variable were the paradigm shifts that opened a new field of inquiry…the Hodgkin–Huxley model-suffices to explain all of the classical properties of action potential excitation and propagation, and even offered a plausible physical basis for the control by membrane potential.” But Hodgkin and Huxley wrote, “The voltage clamp data must not be taken as evidence that our equations are anything more than an empirical description of the time-course of the changes in permeability to sodium and potassium. An equally satisfactory description of the voltage clamp data could no doubt have been achieved with equations of very different form”, and “the success of the equations is no evidence in favour of the mechanism of permeability change that we tentatively had in mind when formulating them”; Hodgkin and Huxley 1952). They seem to be describing normal, not revolutionary science, by addressing questions within the animal electricity paradigm. This does not deprecate their achievements, or that of others who have subsequently determined the mechanisms of electrical signalling (see Armstrong and Hille 1998).

The move from animal spirits to animal electricity resembles a Kuhnian revolution. Growing anomalies in the animal spirit paradigm were emphasised once the alternative of animal electricity was available, but these were initially opposed by ad hoc defences of the animal spirits paradigm. Once accepted, the animal electricity paradigm incommensurably changed the concept of neuronal signalling and the questions asked under a new program of normal science. As with other revolutions it, seems incredible that the animal spirits paradigm was ever believed. However, as Kuhn said, this is from the perspective of the current paradigm and reflects the incommensurability of terms and concepts. It is possible to make links: hydraulic analogies similar to animal spirits are used to explain neuronal electrical signalling to students (Piccolino 1997; Clower 1998). Michael Foster (1924) wrote, “If we judge Descartes from the severe standpoint of exact anatomical knowledge, we are bound to confess that he, to a large extent, introduced a fantastic and unreal anatomy … If we substitute in place of the subtle fluid of the animal spirits, the molecular changes which we call a nervous impulse, if we replace his system of tubes with their valvular arrangements by the present system of concatenated neurons …. Descartes’ exposition will not appear so wholly different from the one which we give today”. Work from Oersted to Hodgkin and Huxley and beyond advanced the animal electricity paradigm by developing new techniques and analyses that allowed novel or better measurements (see Piccolino 1998), but the revolution was the change in concept caused by the animal electricity paradigm, the techniques were developed to address questions under this paradigm, and were aspects of normal science.

## Cortical localisation

Cortical localisation is a dominant paradigm in neuroscience (Zilles and Amunts 2010). That different regions of the brain could serve different functions dates to antiquity (it was mentioned in the Edwin Smith Papyrus dating from 3000BC; see Finger 1994). Hippocrates believed the brain to be the seat of intelligence and madness (based on the work of Alcmaeon), but Aristotle believed that the heart was the seat of rationality, the brain serving to cool the blood. The role of the brain in sensation, rationality and action was ultimately accepted from the work of Galen (because human dissection was forbidden, many of his dissections were done on animals, principally pigs, oxen and Barbary apes; Lanska 2015). The termination of the senses in the brain led Galen to consider that the brain was the seat of rationality, the ventricles being the site of psychic pneuma (Rocca 1997) that caused behaviour. Despite claims by Erasistratos in the third century BC that human intelligence reflected the increased convolutions of the human brain, the Galenic view minimised the role of the cortex (Gross 1987).

The ventricular paradigm persisted for almost 1500 years. It was advanced by Posidonious and Nemesius in the fourth and fifth centuries who placed sensation and imagination in the anterior, reason in the middle, and memory in the posterior ventricles (van der Eijk 2008), a view advanced by medieval scholars (Gross 1987). Human dissections in the fourteenth century initially repeated Galenic dogma, but in the sixteenth century Vesalius revealed over 200 anomalies in Galen’s anatomy, beginning with his “Six Anatomical Tables” in 1538. This included the absence of a rete mirabile at the base of the brain which Galen had identified in animal dissections, and assumed was present in the human brain where it linked the body and the mind (see above; Lanska 2015). However, Galen’s view of the brain persisted despite the anatomical anomalies shown by Vesalius (and while Vesalius was critical of Galen’s anatomy, he adopted Galen’s physiology, including the ventricular paradigm; Gross 2009a, b). The defence of Galen matched aspects that Kuhn outlined of a paradigm in crisis, including persistence through adherence to the scholastic reading of texts rather than direct observation; ad hoc defences (e.g. that the differences Vesalius saw reflected recent anatomical changes, or that missing features were present but invisible); attacks on competence (e.g. that Vesalius was not sufficiently skilled to perform the dissections: this led Vesalius to use animal and human specimens in demonstrations to show his competency in finding the structures Galen had seen in animals, and their absence in humans); or simply refusal to accept evidence by appealing to Galen’s infallibility (see Lanska 2015). Vesalius’s *De Humani Corporis Fabrica* (1543) thus met with harsh criticism from the church and Galenic anatomists. In response he burned the remainder of his unpublished works and preparations for future studies, and lived the rest of his life as a court physician. This has parallels in other challenges to paradigms (e.g. adult neurogenesis; see below).

The view that the cortex was insensitive (cortex is Latin for rind), persisted into the eighteenth century: Albrecht van Haller contrasted the lack of effect of mechanical or chemical stimulation of the cortex with the strong responses evoked by stimulation of deeper brain regions. Emanuel Swedenborg seems to be the first person to consider localisation of functions to specific cortical areas in the eighteenth century (see Gross 1997). Swedenborg correctly located the motor cortex to the precentral gyrus and also correctly claimed it had an inverted somatotopic map. This prescient work had no apparent impact, possibly because Swedenborg lacked a university post, and because his work stopped when he started having religious visions, much of it remaining unpublished until the end of the nineteenth century.

The paradigm shift from ventricular pneuma to cortical localisation was driven by the cranioscopy of Franz Joseph Gall in Vienna at the beginning of the nineteenth century (later named phrenology by Gall’s assistant Johann Spurzheim; Young 1970). Gall claimed one to one correspondences between (1) specific behaviours, (2) an innate faculty, (3) a cortical organ, and (4) cranial prominences caused by cortical growth. He observed 1 and 4, and inferred 2 and 3, generating a mosaic of cranial (and by association cortical) regions associated with specific functions. While Gall’s claims attracted significant public interest, they were not welcomed by the establishment (the Austrian emperor believed that thought was the enemy of stability; see Simpson 2005). As a result Gall left Austria for Paris, where his work, while popular with the public, again met with resistance from the establishment. Napoleon disliked Gall and his views (it is claimed because of Napoleon’s anti-German and anti-materialist views, and because he was dissatisfied with Gall’s reading of his skull; Hedderly 1970). Napoleon pressured the French Academy of Sciences to evaluate Gall’s work. In 1808 Georges Cuvier wrote an unfavourable but not wholly critical report for the Academy. In the 1820s the Academy asked Jean-Pierre Flourens to experimentally examine Gall’s claims. Flourens agreed that Gall’s observations were a useful starting point, but said that experimentation was also needed. Using brain lesions and stimulation by “pricking” the brain with a needle, Flourens showed that the cerebellum was involved in movement, but found little evidence of cortical function (his failures have been attributed to using animals that lacked a well-developed cortex (e.g. pigeons) and his antipathy to cranioscopy; Finger 1994).

Paul Broca is credited with the first example of cortical localisation of function and hemisphere lateralisation in the 1860s from his description of aphasia resulting from a lesion in the left frontal lobe of patient “Tan” (Finger 1994). However, this localisation had been claimed previously by Johann Schmidt in 1673 and Peter Rommel in 1683 who made accurate descriptions of motor aphasia (see Eling and Whitaker 2010); Jean-Baptiste Bouillaud who localised language performance to the same area as Broca using hundreds of examples in the first half of the nineteenth century (his work was largely ignored, possibly because he was an admirer of Gall’s cranioscopy, and because his descriptions were brief); and Marc Dax who suggested in the 1830s that the left hemisphere was specialised for language, discounting Gall’s claim that the two hemispheres were equivalent. Not only was Broca’s claim not novel, but at the start of the twentieth century Pierre Marie suggested that his description of Tan’s lesion was speculative, the brain poorly studied (left hemisphere damage was extensive), and examination of the patient was inadequate (Tan had suffered from epilepsy since youth and had progressive neurological symptoms for 11 years before his death). Rather than a convincing novel piece of work, the acceptance of Broca’s claims may have reflected the desire to reduce the influence of phrenology (see Brown and Chobor 1992; Marshall and Fink 2003).

Ultimate support for cortical localisation was provided by the experimental work of Fritsch and Hitzig in 1870, which showed movements resulting from stimulating regions of the exposed cortex in awake dogs (Young 1970). They also showed that basic movements were unaffected by lesions of the motor cortex that affected movement of the contralateral paw, suggesting, as Swedenborg had (Gross 1997), that voluntary movements were generated by the cortex and more basic movements at lower levels. Fritsch and Hitzig’s work was extended by David Ferrier in dogs and primates. Ferrier outlined multiple areas related to specific aspects of movement, and showed that lesions affected natural movements (Young 1970). Ferrier’s work contrasted with the work of his contemporary Friedrich Goltz, who was unable to abolish function in dogs using cortical lesions. Ferrier and Goltz gave demonstrations at the Seventh International Medical Congress in London in 1881, where Ferrier showed that Goltz’s failure reflected insufficient lesions that spared some sensory and motor function, as well as the reduced cortical dependence of dogs compared to primates, a demonstration that seems to have secured the paradigm status of cortical localisation. Under this paradigm Brodmann made a map of 43 cytoarchitectural areas of the cortex at the start of the twentieth century (see Zilles and Amunts 2010), and stimulation performed during neurosurgery by Wilder Penfield in the mid-twentieth century provided detailed maps of the motor and sensory cortex (Borchers et al. 2012).

Cortical localisation was a paradigm shift that did not reflect any new technique. It incommensurably replaced the paradigm of pneuma contained in ventricles and an unresponsive cortex to become a dominant neuroscience paradigm of cortical localisation of function that provides the basis for experimental investigations (e.g. fMRI, EEG, brain stimulation and lesioning studies), explanations of neurological disorders and surgical interventions, and ubiquitous textbook entries (e.g. cortical maps and homunculi). The trigger for the paradigm shift reflected a complex mix of influences. These included human dissections that highlighted anomalies in Galen’s anatomy (especially the absence of a rete mirabile that Galen said delivered animal spirits to the ventricles; Lanska 2015), although these did not affect the acceptance of Galen’s physiology, suggesting that the anatomical anomalies were of relatively minor importance. The development of the animal electricity paradigm was important as it removed the classical role of the ventricles and pneuma, thus challenging Galen’s physiology and forcing attention onto other mechanisms for generating behaviour. These aspects weakened the adherence to classical ideas of anatomy and physiology. However, the principle trigger for the paradigm shift was Gall’s cranioscopy. Although wrong in detail, this offered an alternative to the ventricular paradigm that Kuhn claimed was necessary for a revolution. Religious and political opposition to Gall’s work due to its supposed promotion of radical views also seems to have contributed. The political aspect is complex. There was growing interest in understanding human behaviour during the enlightenment that was accelerated by revolutionary and social change in the latter part of the seventeenth century that challenged classical and religious authority (Bristow 2017). Although the fixation of character based on brain structure had obvious deterministic associations (phrenology was used to claim inferiority of colonial subjects in Britain), phrenology was popular in France among those on the left and was used to support abolition in the United States (see Staum 2003). The materialist view advanced by Gall’s work was opposed by the church and the conservative establishment in Austria and France. This political opposition prompted the examination of the cortex by Cuvier and Flourens that led to the work of Fritsch and Hitzig, Ferrier and others (Young 1970; Eling and Whitaker 2010), analyses that ultimately led to acceptance of the cortical localisation of function.

## The neuron doctrine and chemical synaptic transmission

The neuron doctrine and chemical transmission are dominant neuroscience paradigms that developed from a pre-paradigm state rather than reflecting paradigm shifts. These are arguably paradigms for some: single cells and the communication between them are important to physiologists, developmental neurobiologists, and molecular neurobiologists, but less important to psychologists who do not typically refer to single neurons (just as biophysicists seldom refer to behaviour). The Golgi stain is acknowledged as crucial for the foundation of the neuron doctrine, and intracellular recordings for chemical synaptic transmission. These may thus offer evidence of techniques driving neuroscience paradigms.

The neuron doctrine is a basic neuroscience paradigm: “no neuroscientific discipline could be understood without recourse to the concept of neuronal individuality and nervous transmission at a synaptic level, as basic units of the nervous system” (Lopez-Munoz et al. 2006). Decades after Schleiden and Schwann had suggested cells are fundamental independent units of tissues, neuroanatomists debated whether this applied to the nervous system. Reticularists saw continuity between elements of the nervous system, while “neuronists” considered these elements were discrete entities (see Shepherd 1991). Reticularists included Held, who identified the calyx synapse in the auditory midbrain, and Gerlach who claimed to have seen fine fibres spreading between neurons in the spinal cord, cortex and cerebellum, but the reticularist view was predominantly associated with Camillo Golgi and his “diffuse nerve network” theory. Waldeyer is credited with the first formulation of the neuron doctrine in 1891, although Ramon y Cajal claimed that he had just popularised evidence obtained by others. This evidence initially came from His’s analyses in the developing spinal cord in the second half of the nineteenth century, and Nansen, and Forel and Gudden in the latter part of the nineteenth century who showed that atrophy of cut nerves was confined to discrete cell groups (see Shepherd 1991). The neuron doctrine is predominantly associated with Santiago Ramon y Cajal due to his forceful defence of the position, his demonstrations that reticular evidence reflected staining artefacts, and his definitive degeneration studies (he wrote, “If neurons were not completely independent, it would be impossible to account for the precise localisation of degeneration following ablation of cell groups or fiber tracts”; Cajal 1995).

The development of the neuron doctrine needed techniques, the Golgi stain was crucial, as of course was the microscope. But these techniques cannot be claimed to have triggered the neuron doctrine paradigm as they were used to support both the neuronist and reticular views. Ramon y Cajal used and improved the Golgi stain in his analyses, but the key advance was his conceptual insight to work on simpler systems rather than human or mammalian tissue as others did, and the use of early developmental stages where the anatomy was simpler. The neuron doctrine also reflected the functional work on spinal reflexes by Charles Sherrington, who named the connection between neurons a “synapse” in Michael Foster’s Textbook of Physiology in 1897. Sherrington saw that reflexes were simpler to explain with separate neurons that allowed spatial and temporal summation, a synaptic delay, and inhibition, all aspects that were difficult to reconcile with a reticular theory. This insight also did not depend on any new tool or technique.

The chemical synaptic transmission paradigm built on the neuron doctrine and Sherrington’s concept of the synapse, and again developed from a pre-paradigm state. In the mid-nineteenth century du Bois-Reymond wrote, “Either there exists at the boundary of the contractile substance a stimulatory secretion … or the phenomenon is electrical in nature” (see Davenport 1991). This dichotomy occupied the first half of the twentieth century. John Newport Langley showed that the physiological effects of nerve stimulation were evoked when nicotine was applied to autonomic ganglia (he showed the same effect on denervated skeletal muscle, an effect blocked by curare; see Davenport 1991). Langley’s student TR Elliot showed that adrenaline evoked similar effects to postganglionic stimulation in the sympathetic nervous system, and thus that “adrenalin might then be the chemical stimulant liberated on each occasion when the impulse arrives at the periphery” (Davenport 1991). Langley concluded that nerve stimulation produces effects “by combining with the receptive substance” (Davenport 1991). This significant claim depended on conceptual insight, not new techniques.

Otto Loewi is credited with demonstrating chemical transmission in 1921. However, Walter Dixon did a similar experiment to Loewi in 1907, showing that an extract from a heart that received vagus stimulation slowed another heart, an effect blocked by atropine: “I interpret these experiments to mean that…when the vagus portion is excited this inhibitory substance is set free”. Dixon didn’t show that this substance was acetylcholine, but Henry Dale in 1914 suggested that adrenaline was released in the sympathetic and acetylcholine in the parasympathetic nervous system (see Valenstein 2005). Rather than reliance on a new technique, Loewi claimed that his heart stimulation experiment came to him in a dream, he went straight to his lab, and had proved chemical transmission by 5.00 a.m. This is revision after the fact: the experiments actually took place over some weeks (Davenport 1991). Loewi’s initial result was difficult to replicate by him and others, and seems to be an example of serendipity in science, in this case due to the experiment being performed in winter: the frog vagus nerve provides inhibitory and excitatory inputs to the heart, the inhibitory cholinergic input that Loewi demonstrated only being significant in winter, while the cooler winter temperature in the lab reduced acetylcholinesterase activity, leaving relatively high low levels of Ach that Loewi could pipette between the two hearts.

Although Langley, Eliot, Dixon and Loewi provided evidence of chemical transmission in the autonomic nervous system, the effects were slow (several hundred ms). This was considered to be acceptable for autonomic functions, but not for central nervous transmission or reflex effects that were an order of magnitude faster. The alternative to chemical transmission that du Bois Reymond had outlined was electrical. John Eccles was the main proponent of this view, often in opposition to Henry Dale. The pre-paradigm status of electrical versus chemical transmission was emphasised by Eccles in a 1936 review in Ergebnisse der Physiologic (he concluded, “At present the chemical and electrical hypotheses must both be regarded as on probation”; see Davenport 1991). The various parasympathetic effects of Ach were termed muscarinic because they were mimicked by muscarine, while preganglionic paraympathetic effects and effects of skeletal muscle were mimicked by nicotine. While the slow muscarinic effects were accepted as chemically-mediated, there was opposition to the fast nicotinic effects being chemical, despite curare blocking muscle responses to nerve stimulation. However, by the mid-1930s chemical transmission at the neuromuscular junction was becoming accepted (Dale 1937). In response, Eccles suggested a compromise of a slow nicotinic chemical component that followed a fast electrical local circuit current from the motor nerve terminal. This was critiqued on theoretical and experimental grounds by Katz and Schmitt (1940). To overcome these objections Eccles suggested that the muscle response reflected an “electroreception” specialisation (see Bennett 2001).

By the end of the 1940s Ralph Gerard and Judith Graham in Chicago had developed glass capillary micropipettes that could be inserted into muscle cells to record intracellular potentials. This wasn’t the first time micropipettes had been used: recordings were made from plant cells at the start of the twentieth century (see Bretag 2017). However, the development of the cathode follower amplifier allowed intracellular recordings of muscle potentials by Fatt and Katz (1951), who showed that changing the potential of the postsynaptic cell altered the endplate potential in ways consistent with the chemical, not electrical hypothesis. However, Eccles still claimed central transmission was electrical, a view promoted by John Fulton, editor of the Journal of Neurophysiology, who wrote in his textbook “Physiology of the Nervous System” (1949), “The idea of a chemical mediator released at the nerve ending and acting directly on the second neurone or muscle thus appears to be unsatisfactory in many respects” (see Todman 2008).

Gilbert Ling, a graduate student of Gerard’s had made micropipettes less than one micrometre at the tip (Bretag 2017). Eccles realised these could be used to record from motor neurons in the cat spinal cord, and with Jack Coombs developed stimulating equipment and amplifiers capable of recording with these high resistance micropipettes. Despite the new and improved recording techniques, the final stage in the electrical versus chemical debate did not reflect these techniques, but Eccles friendship with the philosopher Karl Popper (Todman 2008). Eccles said that Popper encouraged him to state his electrical transmission hypothesis precisely so that it was open to falsification. This hung on inhibition. While electrical excitation could simply reflect depolarisation spreading from a presynaptic to a postsynaptic target, inhibition was difficult to explain electrically. Eccles suggested that “Golgi cells” would cause a biphasic effect of excitation followed by inhibition (Brooks et al. 1948). However, when Eccles and colleagues recorded from motor neurons in the cat spinal cord and stimulated the nerve to antagonist muscles, they saw only inhibitory postsynaptic potentials, something that was “directly opposite to that predicted by the Golgi cell hypothesis, which is thereby falsified”, and “left the chemical hypothesis as the only likely explanation” (Brock et al. 1952). Eccles conceded that if inhibition was chemical then excitation would probably also be, ultimately leading to acceptance of the chemical transmission paradigm. Eccles’ defence of the electrical hypothesis had been so unremitting up until this time that Henry Dale equated his change of view with Saul’s conversion on the road to Damascus (see Davenport 1991).

Chemical transmission is now a major paradigm in neuroscience. Establishing this paradigm depended on intracellular recordings, but Langley, Elliot, and Lowei’s experiments that established the basis for the chemical hypothesis did not depend on a new tool or technique, and Eccles suggests it was Popper’s influence that led him to drop his defence of electrical signalling, not the techniques that he helped to develop and for which he could have claimed significant credit (see Mulkay and Gilbert 1981). The limit of technique in establishing the paradigm is demonstrated by the same techniques being used by Furshpan and Potter (1959) to show electrical excitatory transmission, Furshpan and Furukawa (1962) to show electrically-mediated inhibition, and Martin and Pilar (1963) to show both electrical and chemical transmission at single synapses. It is interesting to speculate how our views of synaptic transmission might been altered if these studies, especially those of Furshpan and Furukawa which would have given a mechanism for electrically-evoked inhibition, had been performed before Eccles et al. had done the work that led to the acceptance of the chemical transmission.

## Adult neurogenesis

A final, recent, example is adult neurogenesis. Once the neuron doctrine was accepted it was believed that no new neurons were added to the adult mammalian brain (Ramón y Cajal 1928; Rakic 1985). This reflected the view that the adult brain was structurally fixed. The “no-new-neurons” paradigm meant that neurogenesis was only considered in the context of the pre or early-postnatal development of the nervous system. Although there were sporadic accounts of adult mammalian neurogenesis during the first half of the twentieth century, it was unclear whether this represented the synthesis of new neurons or other cells (see Gould and Gross 2000).

An important technical advance came with the introduction of ^3^H-thymidine autoradiography as a marker of DNA synthesis in the late 1950s. ^3^H-thymidine can detect DNA replication during cell division (the “S phase”) and thus labels new cells (as it labels any newly synthesised DNA (e.g. during DNA repair) cell division has to be verified; Nowakowski and Hayes 2000). In the 1960s Joseph Altman and colleagues used the ^3^H-thymidine technique in adult rats and cats. In addition to labelling dividing glial cells as expected, they found evidence for neurogenesis in the hippocampal dentate gyrus, the olfactory bulb, and the cerebral cortex (see Altman 2011 for a review of this work). However, despite this evidence, Jacobson (1970) in his textbook on developmental neurobiology wrote “…there is no convincing evidence of neuron production in the brains of adult mammals”, a claim repeated in the second edition (Jacobson 1978). Altman eventually moved away from neurogenesis to focus on other aspects (Altman 2011).

In the 1970s, Michael Kaplan also used ^3^H-thymidine labelling combined with electron microscopy and found evidence of neurogenesis in the adult rat dentate gyrus, olfactory bulb, and visual cortex, supporting Altman’s data, and also showed neurogenesis in the subventricular zone of adult macaque monkeys (some of this work was rejected as being inconclusive and remained unpublished; see Kaplan 2001). Kaplan was inspired to do this work by his undergraduate mentor JW Harper, who was aware of Altman’s data (Kaplan 2001). Negative reaction and failure to get departmental support for further work (despite getting funding) meant that Kaplan, like Altman, left the field (see Kaplan 2001). Kaplan (2001) wrote that, “One of the most fervent supporters of the dogma of no neurogenesis was Pasko Rakic”, and says that data Rakic presented at a conference Kaplan attended in 1984 directly contrasted with Kaplan’s data. Kaplan was a post-doc, Rakic a prominent figure in neuroscience. On asking Rakic if they could discuss their different interpretations, Rakic refused saying that the new cells that Kaplan was showing were not neurons (“those may look like neurons in New Mexico, but they don’t in New Haven”; cited in Specter 2001), and that accepting neurogenesis “would be like removing a page from a book” (cited in Kaplan 2001). Rakic examined adult rhesus monkeys and claimed that neurogenesis only occurred in the prenatal or early postnatal period, and repeated earlier claims that this was necessary for the stability of adult mammalian brain function (Rakic 1985). Rakic’s claims supported the “no-new-neurons” paradigm and his power of authority [and appeal to authority of others (e.g. Ramon y Cajal)] subsequently limited work in the area (Gould 2007).

Acceptance of adult neurogenesis began with studies in adult songbirds in the 1980s by Nottebohm and colleagues (see Nottebohm 1996). Differences in the volume of two song production nuclei that were related to song complexity led Nottebohm et al. to hypothesise, and subsequently show, neurogenesis using ^3^H-thymidine and ultrastructural analyses. Significantly, they showed that the new cells were functionally incorporated into song-related circuits (Nottebohm 1996). This evidence for neurogenesis was readily accepted, but the “no-new-neurons” paradigm was defended by claims that this more primitive system was not representative of mammals (see Specter 2001).

In the 1990s the thymidine analogue 5-bromo-3-deoxyuridine (BrdU) was used to label dividing cells. BrdU labelling was simpler to use and faster than ^3^H-thymidine (see Gould 2007). Immunocytochemical markers that identified labelled cells as neurons or glia were also developed (Gould 2007). Several groups replicated Altman’s and Kaplan’s data using these tools, showing experience-dependent adult neurogenesis in the olfactory bulb, dentate gyrus and neocortex of rats and primates, and also in humans. The weight of this evidence eventually led to the acceptance of adult neurogenesis (see Gould 2007 for review).

Is adult neurogenesis a paradigm shift (Gould and Gross 2000)? The no new neurons paradigm suggested an immutable brain fully formed early in development that influenced ideas about the brain and the normal science done under this paradigm for several decades. It limited questions about neurogenesis to the pre or early postnatal period, and influenced views on the plasticity of the adult brain and how it could be treated after injury or disease. Determining whether something was a revolution or not is difficult, and needs a detailed study of the field either side of the revolution and consideration of who the revolution was for. Adult neurogenesis extends processes underlying neurogenesis, cell migration etc. into adulthood and negates the concept of the brain being complete around birth, thus making it incommensurate with the no-new-neurons paradigm. But does it change our general concepts of the brain? Nowakowski and Hayes (2000) in critiquing the evidence for neurogenesis said that if accepted, neurogenesis would require “re-evaluation of virtually all current conceptual bases for understanding how neuronal circuitries in neocortex develop and are modified”. Neurogenesis has been implicated in memory formation (Kempermann 2008; Aimone et al. 2011), and offers potential neurological repair strategies (Grade and Götz 2017). However, no neurobiologist would deny that practically all neurons are produced during embryogenesis, and general concepts in terms of neuroanatomy and neurophysiology are not obviously challenged by neurogenesis. It would not negate the significance of neurogenesis if it was considered an aspect of normal science done under the neuron doctrine paradigm. However, it seems more likely to be an example of a revolution for some, and one that generates new puzzles of normal science done under this paradigm (e.g. where, how and why neurogenesis occurs, and how it influences function and repair; Fuchs and Flugge 2014).

Kuhn said that during a crisis attempts are made to ignore or dismiss critiques, especially by those strongly associated with the paradigm. It is interesting to consider how the reaction to adult neurogenesis resembles this. Gould and Gross (2000) claimed that Altman’s evidence for adult neurogenesis was ignored because of his junior status. Altman (2011) disputes this: he said he had a faculty position, that many of his opponents were junior to him, and that his work wasn’t ignored. These claims are difficult to verify, but it is a matter of record that he was one of the top 1000 most cited scientists between 1965 and 1978 (Garfield 1981), suggesting his work was acknowledged. However, there was denial: in addition to the quotes from Jacobson above, there was no mention of Altman’s work in Purves and Lichtman’s (1985) textbook on developmental neurobiology, or the third edition of *Principles of Neural Science* (Kandel et al. 1991) which states, “Neurogenesis ceases early in the development of the mammalian brain”. Altman (2011) said that instead of being ignored he was marginalised as funding and publication became difficult. This could be Altman’s attempt to explain his diminishing impact. However, Kaplan (2001) made similar claims. He said that his junior status prevented him from challenging the no new neurons paradigm, wrote about “the political death” of his project, that his “controversial beliefs (were) quashed”, and that those who supported adult neurogenesis “were ignored or silenced.”

Kuhn also said that resistance to change becomes more noticeable during a crisis. The minimal reaction to Altman’s and Kaplan’s work contrasts with the strong and repeated critiques made as evidence for adult neurogenesis grew (see Rayl 1999). Rakic (1998) wrote, “The discovery of neurogenesis in the adult human dentate gyrus is exciting… (but) an exception to the rule of ‘no new neurons’ that still applies to most of the brain”; and Rakic (2002a) “some studies do not satisfy even one basic criterion for neurogenesis”. Rakic (2002b) also appealed to authority: “Kölliker, His and Ramón y Cajal were not only careful scientists, but also profound thinkers—their conclusion that nerve cells…are irreplaceable under normal conditions has so far been correct” [Ramon y Cajal (1928) had said, “Everything may die, nothing may be regenerated”, but added, “It is for the science of the future to change, if possible, this harsh decree”]. In critiquing studies of cortical neurogenesis Rakic (2002b) cited Nowakowski and Hayes’ (2000) critique of Gould et al. (1999), but avoided citing Gould and Gross’ reply, begging the question to defend the paradigm. A paradigm can also defended by attacking the veracity of competing evidence (Rakic’s response to Kaplan), or the competence of researchers and their integrity (it was claimed that in interview Rakic focused on critiquing individuals, especially Gould, rather than their science; see Specter 2001, p. 50). A recent study questioning adult hippocampal neurogenesis in humans (Sorrells et al. 2018; but see Boldrini et al. 2018), quoted Rakic as saying, “I feel vindicated” (see Shen 2018). Why he feels vindicated is unclear given that Rakic replicated evidence for neurogenesis in the adult dentate gyrus (Kornack and Rakic 1999), evidence he said had been “established for decades” (he didn’t cite Altman or Kaplan in association with this claim; Rakic 2002b). In contrast to his extensive critiques of neurogenesis, Rakic’s acceptance of Sorrell et al. illustrates how evidence for or against a paradigm can be evaluated differently. Gage, whose lab showed adult hippocampal neurogenesis (Eriksson et al. 1998), said Sorrells et al. (2018) were, “not really measuring neurogenesis…Neurogenesis is a process, not an event. They just took dead tissue and looked at it at that moment in time” (quoted in Shen 2018).

It is easy to see Rakic as simply blindly defending dogma. Nottebohm said, “we have to keep in mind that he (Rakic) missed this discovery…As much as I hate to say this, I think Pasko Rakic single-handedly held the field of neurogenesis back by at least a decade” (cited in Specter 2001). But assuming no ulterior motive for power or prestige, Rakic was presumably just defending the paradigm that he had worked under. Kuhn said that adherence to a paradigm is necessary to work effectively in a field. Rakic’s role in the neurogenesis debate was not his only contribution to neuroscience. The significant work that he has done presumably required adherence to the no new neurons paradigm that allowed him to address the questions of normal science under this paradigm. Neurogenesis thus illustrates the positive and negative influences of paradigms on scientific progress (Kuhn 1977).

In considering the role of tool development in the acceptance of adult neurogenesis, a combination of techniques were used, beginning with ^3^H-thymidine, electron and confocal microscopy, and BrdU and neuronal and glial markers (Gould and Gross 2000; Gould 2007), as well as human studies using BrdU in cancer patients and C^14^ labelling in postmortem brains (Spalding et al. 2005; Eriksson et al. 1998). While BrdU was important to establishing neurogenesis, similar evidence had been obtained using ^3^H-thymidine autoradiography that did not lead to the paradigm shift. Rather than lack of a tool, the adherence to the “no-new-neurons” paradigm seems to represent a failure of the field to consider anomalous evidence. The crux of the issue was the unequivocal separation of newly generated neurons rather than glia, but Altman was aware of this, showing that satellite (glial) cells lying over a neuron could lead to potential false positives (he used very thin sections to reduce this possibility, and sought evidence that cells were new neurons), while Kaplan used electron microscopy to eliminate artefacts introduced by overlying glial cells. Conversely, as with molecular genetic and optogenetic approaches, the new tools were not a panacea that overcame all previous difficulties. Nowakowski and Hayes (2000) and Gould (2007) highlight how many of the issues and caveats surrounding demonstration of adult neurogenesis remained with the newer techniques. Gould also highlighted how evidence obtained from BrdU can be used to support or negate adult neurogenesis by different people, and even authors in a single paper can interpret the same evidence differently depending on the context (see Gould 2007, pp. 484–485). This “bias” (Gould 2007) presumably reflects differing views due to adherence to particular paradigms.

Various aspects rather than a single key tool thus also seemed to contribute to the neurogenesis paradigm shift. In addition to the work of Gould and Gage et al., the citing of Altman and Kaplan brought these studies back to attention after they had been ignored. The evidence of neurogenesis by Nottebohm and colleagues was readily accepted but considered an exception of simpler systems, arguably served the same purpose as Torpedo in the animal electricity paradigm (see above), by showing that neurogenesis was in principle possible in adult brains. Added to this was the realisation that the adult brain was not immutable, as the argument against neurogenesis used by Ramón y Cajal (1928) and Rakic (1985) claimed, but is subject to considerable plasticity and re-organisation (see Buonomano and Merzenich 1998 for review). This overcomes the conceptual objection of why neurogenesis should occur (acceptance of a plastic rather than fixed adult brain was also a paradigm shift, one not driven by any new technique, and that also met strong opposition by adherents of the previous paradigm; see Taub versus Granit quoted in Schwartz and Begley 2002, pp. 132–148). The use of the newer molecular techniques also weakened the influence of adherents of previous approaches who could claim power of authority in interpreting results (e.g. Rakic’s response to Kaplan’s data; see Specter 2001). These varied influences, together with the growing anomalies against the no new neurons paradigm introduced by newer techniques, eventually caused the paradigm shift to adult neurogenesis.

## Conclusion

Kuhnian terms are frequently used by scientists, but their original meanings are seemingly misunderstood. Consideration of paradigms and crises is useful. For example, does adhering to a paradigm facilitate communication of ideas or does it stifle divergent thinking (the “essential tension”; Kuhn 1977)? Scientists will be familiar with dogma limiting the work they can do or publish, and failing ideas being supported by ad hoc adjustments or attacks on an opponent’s integrity, hence the paraphrasing of Max Plancks aphorism, “science advances one funeral at a time” (Azoulay et al. 2015). In times of crisis a field can close in on itself, especially among the more conservative members who can go to great lengths to stifle challenges or resist change (see Gross 2009a, b; Parker 2006a), and can lead to exaggerated claims (e.g. of causal links between molecules/cells and behaviours when evidence is lacking; Krakauer et al. 2017; Parker 2006b, 2010). Consideration of how paradigms influence scientists or scientific fields is useful given the issues of limited understanding, reproducibility, and translation in neuroscience (e.g. Gilobert and Ovadia 2011; Tsilidis et al. 2013; Ioannidis 2012).

Animal electricity and cerebral localisation match the features of Kuhnian scientific revolutions: growing anomalies in the previous paradigms were initially ignored or resisted, but were emphasised by an alternative view that ultimately triggered a paradigm shift. In none of the neuroscience paradigms discussed here can tool development be claimed as the direct cause of the shift. The animal electricity paradigm needed techniques, but these techniques were developed and used to address questions of normal science once the animal electricity paradigm was accepted. Cortical localisation did not involve any new technique, but reflected a complex mix of anomalies in the previous paradigm and non-scientific influences. The neuron doctrine and chemical transmission developed from pre-paradigm states (reticular vs. neuronal doctrines, and electrical vs. chemical transmission, respectively). New tools were needed, as they were for adult neurogenesis, but in all cases the tools were used to support both sides of the debate and thus cannot claim a key role. None of the paradigms discussed here, which cover a wide range of neuroscience, support the contention that tool development is the key to understanding revolutions in neuroscience.

Maybe the terms scientific revolution and paradigm shift have lost their original meanings by being used in ways that were not intended (e.g. Knafo and Wyart 2015). The OED definition of revolution is “a dramatic or wide-reaching change in conditions, or the state of affairs”. By this definition new tools and techniques can be revolutionary. But just as a political revolutionary does not necessarily cause a political revolution, a revolutionary tool will not necessarily cause a scientific revolution. This needs a conceptual change, reflected in at least some degree of incommensurability with the previous paradigm. While optogenetics and molecular genetics are routinely claimed to have caused revolutions or paradigm shifts, they have so far addressed aspects within existing paradigms, making them elements of normal science. Reasoning is needed with any technique. The impressiveness of a tool is irrelevant if the underlying concepts are wrong (but see Weisberg et al. 2008; McCabe and Castel 2008 for how techniques bias opinions). Bray (2001) writes, “All really big discoveries are the result of thought, in biology as in any other discipline”. New tools allow new analyses that may lead to a revolution, but only if they are put in service of the right questions.
